# Smartphone Application Available for Preventing Group B *Streptococcus* Infections

**Published:** 2014-02-21

Despite more than a decade of prevention efforts and updated prevention guidelines published in 2010, group B *Streptococcus* (GBS) remains the leading cause of early onset neonatal sepsis in the United States (1). A free smartphone application, Prevent Group B Strep, is available from CDC to improve maternal and neonatal management of GBS disease prevention at the point-of-care.

Developed for obstetric and neonatal providers, the GBS prevention application features patient-specific and scenario-specific guidance consistent with the 2010 guidelines for the prevention of perinatal GBS disease (1). The application generates customized user guidance, such as when intrapartum antibiotics are indicated and which antibiotic regimens are appropriate for penicillin-allergic women, based on patient characteristics.

CDC, in collaboration with the American College of Obstetricians and Gynecologists, American Academy of Pediatrics, American College of Nurse-Midwives, and American Academy of Family Physicians, developed the GBS prevention application to improve implementation of evidence-based guidelines and prevent cases of GBS disease. The application is available for Apple iPhone/iPad and Google Android devices. The GBS prevention application and additional information are available at http://www.cdc.gov/groupbstrep/guidelines/prevention-app.html.
